# Supplementary material for: Effects of human mobility on the spread of Dengue in the region of Caldas, Colombia
Source: PLoS Negl Trop Dis. 2023 Nov 27;17(11):e0011087. doi: 10.1371/journal.pntd.0011087 (PMC10703399; doi:10.1371/journal.pntd.0011087)
Supplement: S1 Text — (PDF) [file pntd.0011087.s001.pdf]

Supporting information S1 text

Table A. Impact of increasing geographical distance on the evolution of dengue in a two patches system comprising a node without dengue cases in isolation (zero incidence node) and an endemic node.

Node/parameter	β	λ_m	λ_h	δ_m	μ	N_m	Λ_h	δ_h	N_h
Zero incidence node	0.172	0.408	0.408	0.035	0.329	0	14.554	0.0000278673	396075
Endemic node	0.228	1	0.732	0.029	0.329	2899.52	2.828	0.0000158242	76963

(a) Model parameters

Distance	50.00	100.00	150.00	200.00	250.00	300.00	350.00	400.00	NC
Zero incidence node	59.35	32.08	18.47	11.6	7.84	5.62	4.21	3.26	0.00
Endemic node	16.46	55.17	82.31	97.39	105.88	110.98	114.22	116.41	123.97
Total	75.81	187.25	100.78	108.99	113.73	116.59	118.43	119.675	123.97

(b) Distance between nodes and total number of people infected with dengue by connecting zero incidence node with an endemic node located at different distances. The last row (NC) cases of dengue when the nodes are not connected.

Table B. Impact of increasing geographical distance on the evolution of dengue in a two patches system comprising a node with low incidence in isolation (no endemic node) and an endemic node.

Node/parameter	β	λ_m	λ_h	δ_m	μ	N_m	Λ_h	δ_h	N_h
Endemic node	0.172	0.408	0.732	0.036	0.329	849.76	14.554	0.0000158242	76963
Non endemic node	0.189	0.707	0.729	0.029	0.329	50.66	2.828	0.0000149736	9096

(a) Model parameters

Distance	50.00	100.00	150.00	200.00	250.00	300.00	350.00	400.00	NC
Endemic node	643.93	664.38	668.59	671.01	672.47	673.38	673.98	674.39	675.85
Non endemic node	114.5	94.07	89.71	87.23	85.74	84.81	84.20	83.79	82.31
Total	758.43	758.45	758.3	758.24	758.21	758.19	758.18	758.18	758.16

(b) Distance between nodes and total number of people infected with dengue by connecting endemic node with non-endemic node located at different distances. The last row (NC) cases of dengue when the nodes are not connected.

Table C. Impact of increasing geographical distance on the evolution of dengue in a system of three equidistant patches comprising a node with no dengue cases in isolation (zero incidence node), another one with low incidence (no endemic node) and an endemic node.

Node/parameter	β	λ_m	λ_h	δ_m	μ	N_m	Λ_h	δ_h	N_h
Zero incidence node	0.1717	0.4085	0.408	0.035	0.329	0	14.554	0.0000278673	396075
Endemic node	0.2276	1	0.732	0.029	0.329	2899.52	2.828	0.0000158242	76963
Non endemic node	0.2018	0.9188	0.906	0.032	0.329	2.719	0.2342	0.0000228696	6374

(a) Model parameters

Distance	50	100	150	200	250	300	350	400	NC
Zero incidence node	114.26	64.92	39.45	25.7	17	12.93	9.78	7.64	0.00
Endemic node	30.55	68.18	97.68	117.01	129.24	137.13	142.42	146.09	159.61
No endemic node	23.55	66.55	95.19	110.51	118.9	123.83	126.93	129	136.03
Total	168.36	199.65	229.32	253.22	265.14	273.89	279.13	282.73	295.64

(b) Distance between nodes and total number of people infected with dengue by connecting zero incidence node, endemic node and non endemic node. Located at different distances. The last column (NC) cases of dengue when the nodes are not connected.

Table D. Matrix Υ. Each cell represents the probability that an inhabitant of a municipality moves to another one.

	Aguadas	Anserma	Aranzazu	Belalcazar	Chinchiná	Filadelfía	La Dorada	La Merced	Manizales	Manzanares	Marmato	Marquetalia	Marulanda	Neira	Norcasia
**Aguadas**	0.9000	0.0010	0.0015	0.0003	0.0015	0.0009	0.0009	0.0003	0.0184	0.0011	0.0008	0.0005	0.0004	0.0020	0.0002
**Anserma**	0.0004	0.9000	0.0003	0.0018	0.0052	0.0005	0.0005	0.0004	0.0300	0.0003	0.0005	0.0001	0.0001	0.0015	0.0000
**Aranzazu**	0.0013	0.0007	0.9000	0.0002	0.0028	0.0094	0.0005	0.0014	0.0479	0.0003	0.0008	0.0001	0.0002	0.0096	0.0000
**Belalcazar**	0.0003	0.0055	0.0003	0.9000	0.0058	0.0003	0.0005	0.0003	0.0333	0.0003	0.0004	0.0001	0.0000	0.0016	0.0000
**Chinchiná**	0.0001	0.0006	0.0001	0.0002	0.9000	0.0001	0.0001	0.0001	0.0448	0.0001	0.0001	0.0000	0.0000	0.0011	0.0000
**Filadelfía**	0.0007	0.0010	0.0085	0.0002	0.0026	0.9000	0.0004	0.0057	0.0459	0.0002	0.0014	0.0001	0.0001	0.0098	0.0000
**La Dorada**	0.0006	0.0010	0.0004	0.0003	0.0023	0.0004	0.9000	0.0002	0.0217	0.0039	0.0002	0.0046	0.0002	0.0014	0.0057
**La Merced**	0.0007	0.0020	0.0033	0.0005	0.0027	0.0149	0.0005	0.9000	0.0336	0.0002	0.0021	0.0001	0.0002	0.0053	0.0000
**Manizales**	0.0001	0.0005	0.0003	0.0002	0.0065	0.0004	0.0002	0.0001	0.9000	0.0001	0.0001	0.0001	0.0000	0.0062	0.0000
**Manzanares**	0.0013	0.0009	0.0004	0.0003	0.0024	0.0004	0.0064	0.0001	0.0266	0.9000	0.0002	0.0183	0.0017	0.0015	0.0002
**Marmato**	0.0021	0.0036	0.0023	0.0009	0.0050	0.0046	0.0008	0.0027	0.0310	0.0004	0.9000	0.0002	0.0002	0.0017	0.0000
**Marquetalia**	0.0009	0.0007	0.0003	0.0002	0.0018	0.0003	0.0119	0.0001	0.0180	0.0293	0.0001	0.9000	0.0006	0.0011	0.0004
**Marulanda**	0.0041	0.0016	0.0022	0.0003	0.0026	0.0014	0.0034	0.0009	0.0282	0.0162	0.0007	0.0039	0.9000	0.0030	0.0002
**Neira**	0.0002	0.0003	0.0010	0.0001	0.0022	0.0011	0.0002	0.0002	0.0855	0.0001	0.0001	0.0000	0.0000	0.9000	0.0000
**Norcasia**	0.0014	0.0006	0.0002	0.0002	0.0012	0.0002	0.0557	0.0001	0.0113	0.0011	0.0001	0.0015	0.0001	0.0007	0.9000
**Pacora**	0.0540	0.0007	0.0019	0.0002	0.0016	0.0010	0.0008	0.0008	0.0188	0.0011	0.0005	0.0005	0.0004	0.0022	0.0001
**Palestina**	0.0001	0.0007	0.0001	0.0003	0.0637	0.0001	0.0001	0.0000	0.0272	0.0001	0.0001	0.0000	0.0000	0.0008	0.0000
**Pensilvania**	0.0013	0.0010	0.0004	0.0003	0.0026	0.0004	0.0067	0.0001	0.0268	0.0336	0.0002	0.0078	0.0009	0.0016	0.0003
**Riosucio**	0.0006	0.0114	0.0010	0.0006	0.0024	0.0018	0.0004	0.0010	0.0150	0.0002	0.0013	0.0001	0.0001	0.0019	0.0000
**Risaralda**	0.0002	0.0322	0.0002	0.0038	0.0051	0.0004	0.0003	0.0002	0.0269	0.0002	0.0002	0.0001	0.0000	0.0012	0.0000
**Salamina**	0.0047	0.0017	0.0112	0.0003	0.0029	0.0033	0.0007	0.0040	0.0390	0.0013	0.0011	0.0005	0.0006	0.0056	0.0000
**Samaná**	0.0010	0.0055	0.0004	0.0003	0.0023	0.0004	0.0191	0.0001	0.0218	0.0075	0.0002	0.0112	0.0005	0.0014	0.0042
**San José**	0.0003	0.0047	0.0003	0.0223	0.0049	0.0004	0.0004	0.0002	0.0268	0.0002	0.0003	0.0001	0.0000	0.0012	0.0000
**Supía**	0.0004	0.0034	0.0009	0.0004	0.0020	0.0020	0.0002	0.0012	0.0118	0.0001	0.0014	0.0001	0.0001	0.0017	0.0000
**Victoria**	0.0005	0.0006	0.0002	0.0002	0.0014	0.0002	0.0252	0.0001	0.0144	0.0058	0.0001	0.0135	0.0003	0.0009	0.0006
**Villamaría**	0.0000	0.0001	0.0000	0.0000	0.0008	0.0000	0.0000	0.0000	0.0979	0.0000	0.0000	0.0000	0.0000	0.0005	0.0000
**Viterbo**	0.0003	0.0152	0.0004	0.0100	0.0038	0.0004	0.0006	0.0002	0.0332	0.0003	0.0003	0.0002	0.0000	0.0010	0.0000
**Honda**	0.0000	0.0000	0.0000	0.0000	0.0000	0.0000	0.0898	0.0000	0.0000	0.0000	0.0000	0.0000	0.0000	0.0000	0.0000
**Marsella**	0.0000	0.0000	0.0000	0.0000	0.0753	0.0000	0.0000	0.0000	0.0000	0.0000	0.0000	0.0000	0.0000	0.0000	0.0000
**Santa Rosa de cabal**	0.0000	0.0000	0.0000	0.0000	0.1000	0.0000	0.0000	0.0000	0.0000	0.0000	0.0000	0.0000	0.0000	0.0000	0.0000
**Jardín**	0.0000	0.0000	0.0000	0.0000	0.0000	0.0000	0.0000	0.0000	0.0000	0.0000	0.0000	0.0000	0.0000	0.0000	0.0000
**La pintada**	0.0870	0.0000	0.0000	0.0000	0.0000	0.0000	0.0000	0.0000	0.0000	0.0000	0.0000	0.0000	0.0000	0.0000	0.0000
**Sonson**	0.0963	0.0000	0.0000	0.0000	0.0000	0.0000	0.0000	0.0000	0.0000	0.0000	0.0000	0.0000	0.0000	0.0000	0.0037

	Pacora	Palestina	Pensilvania	Riosucio	Risaralda	Salamina	Samaná	San José	Supía	Victoria	Villamaría	Viterbo	Honda	Marsella	Santa Rosa de cabal	Jardín	La pintada	Sonson
**Aguadas**	0.0371	0.0005	0.0008	0.0031	0.0003	0.0047	0.0005	0.0002	0.0017	0.0002	0.0023	0.0003	0.0000	0.0000	0.0000	0.0000	0.0023	0.0161
**Anserma**	0.0002	0.0023	0.0002	0.0231	0.0161	0.0006	0.0010	0.0015	0.0050	0.0001	0.0038	0.0046	0.0000	0.0000	0.0000	0.0000	0.0000	0.0000
**Aranzazu**	0.0011	0.0008	0.0002	0.0043	0.0003	0.0094	0.0002	0.0002	0.0029	0.0001	0.0051	0.0002	0.0000	0.0000	0.0000	0.0000	0.0000	0.0000
**Belalcazar**	0.0002	0.0026	0.0002	0.0037	0.0061	0.0003	0.0002	0.0221	0.0017	0.0001	0.0043	0.0095	0.0000	0.0000	0.0000	0.0000	0.0000	0.0000
**Chinchiná**	0.0000	0.0230	0.0001	0.0006	0.0003	0.0001	0.0000	0.0002	0.0003	0.0000	0.0047	0.0001	0.0000	0.0018	0.0212	0.0000	0.0000	0.0000
**Filadelfía**	0.0005	0.0008	0.0002	0.0073	0.0004	0.0025	0.0001	0.0003	0.0060	0.0001	0.0048	0.0003	0.0000	0.0000	0.0000	0.0000	0.0000	0.0000
**La Dorada**	0.0004	0.0007	0.0029	0.0015	0.0003	0.0005	0.0063	0.0002	0.0007	0.0066	0.0032	0.0003	0.0336	0.0000	0.0000	0.0000	0.0000	0.0000
**La Merced**	0.0011	0.0008	0.0002	0.0109	0.0006	0.0078	0.0001	0.0004	0.0095	0.0001	0.0022	0.0004	0.0000	0.0000	0.0000	0.0000	0.0000	0.0000
**Manizales**	0.0001	0.0014	0.0001	0.0005	0.0002	0.0002	0.0001	0.0001	0.0003	0.0000	0.0819	0.0002	0.0000	0.0000	0.0000	0.0000	0.0000	0.0000
**Manzanares**	0.0009	0.0008	0.0232	0.0012	0.0003	0.0015	0.0040	0.0002	0.0006	0.0025	0.0039	0.0003	0.0000	0.0000	0.0000	0.0000	0.0000	0.0000
**Marmato**	0.0008	0.0016	0.0003	0.0181	0.0007	0.0028	0.0003	0.0007	0.0143	0.0001	0.0041	0.0007	0.0000	0.0000	0.0000	0.0000	0.0000	0.0000
**Marquetalia**	0.0006	0.0006	0.0086	0.0010	0.0002	0.0010	0.0097	0.0002	0.0005	0.0092	0.0026	0.0002	0.0000	0.0000	0.0000	0.0000	0.0000	0.0000
**Marulanda**	0.0033	0.0008	0.0060	0.0045	0.0003	0.0062	0.0023	0.0002	0.0024	0.0011	0.0035	0.0004	0.0000	0.0000	0.0000	0.0000	0.0000	0.0000
**Neira**	0.0001	0.0006	0.0001	0.0009	0.0001	0.0005	0.0001	0.0001	0.0006	0.0000	0.0060	0.0001	0.0000	0.0000	0.0000	0.0000	0.0000	0.0000
**Norcasia**	0.0006	0.0004	0.0013	0.0009	0.0001	0.0003	0.0138	0.0001	0.0004	0.0014	0.0016	0.0002	0.0000	0.0000	0.0000	0.0000	0.0000	0.0042
**Pacora**	0.9000	0.0005	0.0008	0.0019	0.0003	0.0075	0.0004	0.0001	0.0010	0.0002	0.0023	0.0002	0.0000	0.0000	0.0000	0.0000	0.0000	0.0000
**Palestina**	0.0000	0.9000	0.0001	0.0005	0.0004	0.0001	0.0000	0.0002	0.0003	0.0000	0.0032	0.0002	0.0000	0.0016	0.0000	0.0000	0.0000	0.0000
**Pensilvania**	0.0009	0.0009	0.9000	0.0015	0.0003	0.0014	0.0036	0.0003	0.0007	0.0019	0.0039	0.0004	0.0000	0.0000	0.0000	0.0000	0.0000	0.0000
**Riosucio**	0.0002	0.0008	0.0002	0.9000	0.0015	0.0012	0.0001	0.0007	0.0507	0.0001	0.0020	0.0010	0.0000	0.0000	0.0000	0.0037	0.0000	0.0000
**Risaralda**	0.0002	0.0025	0.0002	0.0060	0.9000	0.0002	0.0001	0.0093	0.0016	0.0000	0.0034	0.0051	0.0000	0.0000	0.0000	0.0000	0.0003	0.0000
**Salamina**	0.0051	0.0009	0.0008	0.0064	0.0003	0.9000	0.0005	0.0002	0.0041	0.0001	0.0045	0.0002	0.0000	0.0000	0.0000	0.0000	0.0000	0.0000
**Samaná**	0.0006	0.0007	0.0047	0.0015	0.0003	0.0010	0.9000	0.0002	0.0007	0.0108	0.0032	0.0003	0.0000	0.0000	0.0000	0.0000	0.0000	0.0000
**San José**	0.0001	0.0023	0.0002	0.0047	0.0149	0.0003	0.0001	0.9000	0.0014	0.0001	0.0034	0.0103	0.0000	0.0000	0.0000	0.0000	0.0000	0.0000
**Supía**	0.0002	0.0006	0.0001	0.0695	0.0005	0.0010	0.0001	0.0003	0.9000	0.0000	0.0015	0.0004	0.0000	0.0000	0.0000	0.0000	0.0000	0.0000
**Victoria**	0.0003	0.0005	0.0030	0.0008	0.0002	0.0003	0.0136	0.0001	0.0004	0.9000	0.0021	0.0002	0.0146	0.0000	0.0000	0.0000	0.0000	0.0000
**Villamaría**	0.0000	0.0002	0.0000	0.0001	0.0000	0.0000	0.0000	0.0000	0.0000	0.0000	0.9000	0.0000	0.0000	0.0000	0.0000	0.0000	0.0000	0.0000
**Viterbo**	0.0001	0.0025	0.0003	0.0070	0.0084	0.0003	0.0002	0.0107	0.0021	0.0001	0.0023	0.9000	0.0000	0.0000	0.0000	0.0000	0.0000	0.0000
**Honda**	0.0000	0.0000	0.0000	0.0000	0.0000	0.0000	0.0000	0.0000	0.0000	0.0102	0.0000	0.0000	0.9000	0.0000	0.0000	0.0000	0.0000	0.0000
**Marsella**	0.0000	0.0247	0.0000	0.0000	0.0000	0.0000	0.0000	0.0000	0.0000	0.0000	0.0000	0.0000	0.0000	0.9000	0.0000	0.0000	0.0000	0.0000
**Santa Rosa de cabal**	0.0000	0.0000	0.0000	0.0000	0.0000	0.0000	0.0000	0.0000	0.0000	0.0000	0.0000	0.0000	0.0000	0.0000	0.9000	0.0000	0.0000	0.0000
**Jardín**	0.0000	0.0000	0.0000	0.1000	0.0000	0.0000	0.0000	0.0000	0.0000	0.0000	0.0000	0.0000	0.0000	0.0000	0.0000	0.9000	0.0000	0.0000
**La pintada**	0.0000	0.0000	0.0000	0.0000	0.0130	0.0000	0.0000	0.0000	0.0000	0.0000	0.0000	0.0000	0.0000	0.0000	0.0000	0.0000	0.9000	0.0000
**Sonson**	0.0000	0.0000	0.0000	0.0000	0.0000	0.0000	0.0000	0.0000	0.0000	0.0000	0.0000	0.0000	0.0000	0.0000	0.0000	0.0000	0.0000	0.9000

Table E. Estimated values of the model parameters for each municipality

Municipality	$\delta_h(\times 10^{-5})$	β	λ_m	λ_h	δ_m	μ	Λ_h	Λ_m min	Λ_m max
Aguadas	2.55	0.17	0.44	0.45	0.04	0.329	0	0	0
Anserma	3.00	0.18	0.51	0.52	0.04	0.329	36.7	30	300
Aranzazu	2.30	0.17	0.41	0.41	0.03	0.329	0	0	0
Belalcazar	1.80	0.18	0.57	0.58	0.04	0.329	19.45	13.66	147.36
Chinchiná	2.52	0.18	0.58	0.59	0.04	0.329	50.88	48.63	533.08
Filadelfía	3.45	0.18	0.55	0.57	0.04	0.329	7.45	2	83.3
La Dorada	1.58	0.23	1.36	0.73	0.03	0.329	354.7	86.6	821.3
La Merced	3.67	0.19	0.77	0.79	0.03	0.329	0	0	0
Manizales	2.79	0.18	0.55	0.56	0.04	0.329	0	0	0
Manzanares	2.49	0.18	0.63	0.65	0.04	0.329	0	0	0
Marmato	1.49	0.19	0.71	0.73	0.04	0.329	19.25	15.88	277.58
Marquetalia	2.26	0.18	0.59	0.61	0.04	0.329	48.9	33.9	339
Marulanda	3.34	0.15	0.04	0.04	0.05	0.329	0	0	0
Neira	2.92	0.17	0.41	0.41	0.03	0.329	29.66	3.65	40.72
Norcasia	2.29	0.2	0.92	0.91	0.03	0.329	7.16	6.32	112.71
Pacora	2.84	0.17	0.44	0.44	0.04	0.329	0	0	0
Palestina	4.50	0.19	0.69	0.71	0.04	0.329	19.872	9.3	497
Pensilvania	2.85	0.18	0.53	0.54	0.04	0.329	3.46	1.65	36.5
Riosucio	2.64	0.18	0.57	0.58	0.04	0.329	13.94	3.94	139.38
Risaralda	2.67	0.18	0.48	0.49	0.04	0.329	0	0	0
Salamina	3.37	0.19	0.70	0.72	0.04	0.329	0	0	0
Samaná	2.76	0.18	0.54	0.55	0.04	0.329	16.9	6.9	169
San José	2.62	0.17	0.41	0.41	0.03	0.329	14.41	4.41	44.1
Supía	2.07	0.187	0.67	0.69	0.04	0.329	16.03	5.64	204.57
Victoria	2.66	0.21	0.99	0.95	0.03	0.329	5.69	4.39	44
Villamaría	2.24	0.17	0.41	0.41	0.03	0.32	6.58	3.65	36.5
Viterbo	2.52	0.19	0.85	0.85	0.03	0.35	23.73	15.71	308.05

Table F. Average altitude and temperature of the municipalities of the department of Caldas. Source: Caldas Health Observatory Indicator Booklets. [1].

Municipality	Altitude [masl]	T Average [$^{\circ}C$]	Municipality	Altitude [masl]	T Average [$^{\circ}C$]	Municipality	Altitude [masl]	T Average [$^{\circ}C$]
Aguadas	2214	18.5	Manzanares	1871	21.1	Riosucio	1380	20.2
Anserma	1720	19.4	Marmato	1370	22.1	Risaralda	1743	19
Aranzazu	1960	18	Marquetalia	1600	20.5	Salamina	1775	22
Belalcazar	1632	20.2	Marulanda	2825	13	Samaná	1460	19.8
Chinchiná	1380	20.3	Neira	1969	18	San José	1710	18
Filadelfía	1620	20	Norcasia	700	25	Supía	1183	21.6
La Dorada	178	31	Pacora	1819	18.4	Victoria	710	26
La Merced	1819	23	Palestina	1630	21.8	Villamaría	1920	18
Manizales	2150	19.9	Pensilvania	2100	19.7	Viterbo	998	24

Table G. Model initial conditions for each municipality.

Municipality	$S_m(0)$	$I_{mRain}(0)$	$I_{mDrynees}(0)$	$S_h(0)$	$I_h(0)$	$R_h(0)$
Aguadas	0	0	0.001	22081	0	0
Anserma	37.704	2.94	0.001	33792	0	0
Aranzazu	0	0	0.001	11422	0	0
Belalcazar	17.167	1.45	0.001	10863	0	0
Chinchiná	38.763	5.8	0.001	51492	0	0
Filadelfía	5.553	0.11	0.001	11034	0	0
La Dorada	57.411	3.3	0.001	76963	0	0
La Merced	0	0	0.001	5508	0	0
Manizales	0	38	0.001	396075	0	0
Manzanares	0	0	0.001	23274	0	0
Marmato	165.579	21.5	0.001	9096	0	0
Marquetalia	18.520	1.45	0.001	14992	0	0
Marulanda	0	0	0.001	3406	0	0
Neira	3.713	0.193	0.001	30513	0	0
Norcasia	62.603	9.5	0.001	6374	0	0
Pácora	0	0	0.001	11952	0	0
Palestina	79.036	9.85	0.001	17760	0	0
Pensilvania	50.405	5.3	0.001	26361	0	0
Riosucio	34.613	3	0.001	61535	0	0
Risaralda	0	0	0.001	9583	0	0
Salamina	0	0	0.001	16635	0	0
Samaná	63.076	4.85	0.001	25777	0	0
San José	5.549	0.2	0.001	7588	0	0
Supía	14.036	2.4	0.001	26728	0	0
Victoria	5.253	0.8	0.001	8415	0	0
Villamaría	9.283	1.3	0.001	56303	0	0
Viterbo	10.244	1.4	0.001	12469	0	0

Table H. Total number of dengue cases in the municipalities of Caldas obtained through the calibration of the proposed model.

Municipality	# cases	Municipality	# cases	Municipality	# cases
Aguadas	1	Manzanares	2	Riosucio	25
Anserma	16	Marmato	265	Risaralda	1
Aranzazu	1	Marquetalia	10	Salamina	1
Belalcazar	8	Marulanda	0.0	Samaná	32
Chinchiná	60	Neira	2	San José	2
Filadelfía	2	Norcasia	170	Supía	39
La Dorada	125	Pacora	0.0	Victoria	13
La Merced	1	Palestina	48	Villamaría	5
Manizales	149	Pensilvania	26	Viterbo	29
Total	1033				

Table I. Dengue incidence rate per 100 000 inhabitants, by municipality of origin. Source: Public Health Observatory [1].

Municipality	**2015**	**2016**	**2017**	**2018**	**2019**	**2020**	Total Municipality
Marmato	2836.4	571.7	120.9	55.0	77.0	22.0	3682.9
Norcasia	2682.8	47.1	0.0	31.4	627.5	31.4	3420.1
Viterbo	248.6	200.5	24.1	0.0	312.8	24.1	810.0
Risaralda	20.9	427.8	41.7	20.9	0.0	167.0	678.3
La Dorada	157.2	46.8	7.8	18.2	283.3	52.0	565.2
Supía	138.4	396.6	7.5	3.7	7.5	3.7	557.5
Palestina	264.6	163.3	28.2	5.6	39.4	28.2	529.3
Chinchiná	112.6	324.3	33.0	5.8	21.4	25.2	522.4
Victoria	83.2	11.9	11.9	23.8	118.8	154.5	404.0
Marquetalia	73.4	186.8	33.4	0.0	66.7	13.3	373.5
Filadelfía	9.1	308.1	0.0	0.0	0.0	0.0	317.2
Samaná	120.3	42.7	3.9	11.6	42.7	7.8	228.9
Anserma	50.3	115.4	3.0	0.0	0.0	8.9	177.6
Riosucio	37.4	134.9	1.6	0.0	1.6	1.6	177.1
Aguadas	122.3	13.6	27.2	0.0	0.0	0.0	163.0
Belalcazar	73.6	46.0	0.0	0.0	0.0	9.2	128.9
Pensilvania	98.6	19.0	0.0	3.8	0.0	0.0	121.4
Salamina	18.0	72.1	6.0	0.0	0.0	6.0	102.2
La Merced	36.3	36.3	0.0	0.0	0.0	0.0	72.6
Manizales	38.1	2.3	3.0	0.3	0.0	1.5	45.2
Villamaría	7.1	14.2	3.6	0.0	1.8	0.0	26.6
San José	13.2	13.2	0.0	0.0	0.0	0.0	26.4
Neira	3.3	3.3	3.3	0.0	0.0	3.3	13.1
Pácora	8.4	0.0	0.0	0.0	0.0	0.0	8.4
Manzanares	0.0	0.0	0.0	0.0	4.3	0.0	4.3
Aranzazu	0.0	0.0	0.0	0.0	0.0	0.0	0.0
Marulanda	0.0	0.0	0.0	0.0	0.0	0.0	0.0

Table J. Dengue cases in each of the 5 analyzed municipalities when applying the three mobility restrictions. Column five shows the application of the mobility restriction in all 5 municipalities simultaneously.

				Full connection - non access to						Full connection - isolated						Full connection - Non exit					
**Municipality**	**Real cases**	**Unrestricted mobility**	**Restricted mobility**	**La Dorada**	**Norcasia**	**Marmato**	**Manizales**	**Chinchiná**	**five**	**La Dorada**	**Norcasia**	**Marmato**	**Manizales**	**Chinchiná**	**five**	**La Dorada**	**Norcasia**	**Marmato**	**Manizales**	**Chinchiná**	**five**
Aguadas	27	1	0	1	1	1	1	1	0	1	1	1	1	1	0	1	1	1	1	1	1
Anserma	17	16	15	16	16	16	16	16	15	17	16	16	16	16	15	17	16	16	17	17	17
Aranzazu	0	1	0	1	1	0	0	1	0	1	1	0	0	1	0	1	1	1	1	1	1
Belalcazar	8	8	8	8	8	8	8	8	8	8	8	8	8	8	8	8	8	8	8	8	8
Chinchina	58	60	78	60	60	60	60	84	83	61	60	60	64	78	78	61	60	60	64	56	59
Filadelfia	1	2	1	2	2	1	1	2	1	2	2	1	1	2	1	2	2	2	2	2	2
La Dorada	121	125	63	118	106	124	124	125	96	63	99	124	124	125	63	73	119	125	125	125	68
La Merced	2	1	0	1	1	0	1	1	0	1	1	0	1	1	0	1	1	1	1	1	1
Manizales	151	149	149	149	149	148	157	146	153	150	149	148	149	147	149	150	149	149	141	150	143
Manzanares	0	2	0	2	2	2	2	2	1	2	2	2	2	2	1	2	2	2	2	2	2
Marmato	258	265	286	265	265	287	265	265	287	266	265	286	267	265	286	266	265	266	267	265	269
Marquetalia	11	10	10	10	10	10	10	10	10	10	10	10	10	10	10	11	10	10	10	10	11
Marulanda	0	0	0	0	0	0	0	0	0	0	0	0	0	0	0	0	0	0	0	0	0
Neira	1	2	1	2	2	2	1	2	1	2	2	2	1	2	1	2	2	2	2	2	2
Norcasia	171	170	226	172	227	170	170	170	226	201	226	170	170	170	226	200	173	170	170	170	202
Pacora	1	0	0	0	0	0	0	0	0	0	0	0	0	0	0	0	0	0	0	0	0
Palestina	47	48	56	48	48	48	48	47	46	48	48	48	50	50	52	48	48	48	50	51	54
Pensilvania	26	26	25	25	25	25	25	26	25	26	25	25	25	26	25	26	26	26	26	26	26
Riosucio	23	25	17	25	25	22	24	24	21	25	25	22	24	24	21	25	25	24	25	25	24
Risaralda	2	1	0	1	1	1	1	1	0	1	1	1	1	1	0	1	1	1	1	1	1
Salamina	3	1	0	1	1	1	1	1	0	1	1	1	1	1	0	1	1	1	1	1	1
Samana	31	32	28	31	29	32	32	32	28	33	29	32	32	32	28	33	32	32	32	32	33
San Jose	1	2	1	2	2	1	1	2	1	2	2	1	1	2	1	2	2	2	2	2	2
Supia	37	39	45	39	39	37	39	39	37	39	39	36	39	39	36	39	39	38	39	39	38
Victoria	7	13	13	13	13	13	13	13	12	14	13	13	13	13	14	15	13	13	13	13	15
Villamaria	4	5	5	5	5	5	3	5	3	5	5	5	5	5	5	5	5	5	7	5	7
Viterbo	31	29	31	29	29	29	29	29	0	29	29	29	29	29	29	29	29	29	29	29	29
Total	1039	1033	1058	1025	1066	1044	1033	1051	1055	1006	1057	1041	1035	1049	1051	1018	1029	1031	1036	1034	1015

Table K. Dengue cases with and without mobility restrictions. Negative (positive) numbers mean a reduction (increase) in the number of cases after enforcing quarantine in the population, thus removing the mobility network.

Municipality	Unrestricted mobility	Restricted mobility	Variation in dengue cases	% of variation
La Dorada	125	63	-62	-50
Riosucio	25	17	-8	-32
Samana	32	28	-4	-13
Manzanares	2	0	-2	-100
Anserma	16	15	-1	-6
Neira	2	1	-1	-50
Aguadas	1	0	-1	-100
Salamina	1	0	-1	-100
Filadelfia	2	1	-1	-50
Aranzazu	1	0	-1	-100
Risaralda	1	0	-1	-100
La Merced	1	0	-1	-100
San Jose	2	1	-1	-50
Belalcazar	8	8	0	0
Pacora	0	0	0	0
Pensilvania	26	25	-1	-4
Marquetalia	10	10	0	0
Villamaria	5	5	0	0
Marulanda	0	0	0	0
Manizales	149	149	0	0
Victoria	13	13	0	0
Viterbo	29	31	2	7
Supia	39	45	6	15
Palestina	48	56	8	17
Chinchina	60	78	18	30
Marmato	265	286	21	8
Norcasia	170	226	56	33
total	1033	1058	25	2

Table L. Percentage of travelers going from each municipality to either zero incidence nodes or areas presenting lower or higher incidence. Flows to areas included in the mobility matrix but without incidence data are not included in this table.

Municipality	Incidence when mobility is restricted	% of population traveling to municipalities with higher incidence	% of population traveling to municipalities with lower incidence	% of population traveling to municipalities of with zero incidence
Aguadas	0.00	3.62	0.00	4.55
Anserma	44.39	2.13	6.03	1.83
Aranzazu	0.00	8.62	0.00	1.38
Belalcazar	73.64	2.12	7.10	0.78
Chinchiná	151.48	2.36	5.26	0.08
Filadelfía	9.06	6.67	1.46	1.87
La Dorada	81.86	2.57	3.42	0.65
La Merced	0.00	8.62	0.00	1.38
Manizales	37.62	0.96	8.91	0.13
Manzanares	0.00	9.38	0.00	0.62
Marmato	3144.24	0.00	8.80	1.19
Marquetalia	66.7	4.32	2.38	3.30
Marulanda	0.00	6.66	0.00	3.34
Neira	3.28	9.78	0.00	0.22
Norcasia	3545.65	0.00	9.18	0.40
Pácora	0.00	3.39	0.00	6.61
Palestina	315.32	0.01	9.75	0.08
Pensilvania	94.84	1.07	5.03	3.90
Riosucio	27.63	8.41	0.65	0.57
Risaralda	0.00	9.85	0.00	0.13
Salamina	0.00	7.28	0.00	2.72
Samaná	108.62	1.93	6.93	1.14
San José	13.18	7.86	0.51	1.63
Supía	168.36	0.25	9.30	0.45
Victoria	154.49	0.17	7.60	0.77
Villamaría	8.88	9.93	0.05	0.02
Viterbo	248.62	0.29	8.70	1.01

References

1. Publica S. Cartilla para el diligenciamiento de la ficha familiar. Territorial de salud de Caldas; 2015.
