# Supplementary material for: Effects of human mobility on the spread of Dengue in the region of Caldas, Colombia
Source: PLoS Negl Trop Dis. 2023 Nov 27;17(11):e0011087. doi: 10.1371/journal.pntd.0011087 (PMC10703399; doi:10.1371/journal.pntd.0011087)
Supplement: Attachment — Submitted filename: Comments.pdf [file pntd.0011087.s002.pdf]

-Effects of human mobility on the spread of Dengue in the region of Caldas, Colombia.

The article provides relevant information about the comprehension of dengue dynamics in Colombia, using modeling techniques and suggesting measures to control the spread of the disease considering human mobility strategies.

Dear authors:

Please consider explaining why the simulation time of 400 days was chosen.

Is there any stabilization when running the model for some more years?, dengue presents seasonality associated with temperature and rainy seasons, this was considered on the model (page 11), but it is not possible to observe it clearly on the general modeling results (Fig. 5), maybe at the final of the simulation. This effect seems to be present on the likelihood of a person becoming infectious (Fig.1 d). Maybe running for some more years this could become evident. Also, consider that a seasonal pattern could be present at holidays when people move across different regions.

Page 6. Section 2.1. Parameters are time dependent, consider including the time scale used. I suppose they are days.

Page 6. Section 2.2. Which are the considerations or references behind the assumption that 10% of the people are moving to another area and which is the time frame of the assumption.

Page 6. Figure 1c and 1f. Consider moving the legend from above the result.

Equation 11. Please consider checking if in the compartments Sm*i*, Im*i*, Shi and Ih*i*, *the notation "he" was* defined previously. I suppose it is the human mobility flows integration, e from effective population. This could be added to the parameters table.

Page 8. On Fig. 3 please consider increasing the differentiation thickness of lines (*ik, ki), (ki, kj) (ji ij) (kj jk)* or try a combination of color patches to get an even better understanding of the meta-population dynamics.

Page 10. Figure 5c. It is not possible to see the total number of cases.

Page 10. section 3.1.3 space is lacking in "locally,the situation".

Table 5 how the prob were calculated.

Table 6. Consider indicating that this is a model prediction of Dengue at full connection.

Page 11, last paragraph please consider rephrasing for better understanding: “The agreement between real cases and those obtained after the SIR-SI model has been calibrated is shown in Fig.”.

I hope these suggestions will help improve the manuscript and the publication process.
