# Supplementary material for: Effects of human mobility on the spread of Dengue in the region of Caldas, Colombia
Source: PLoS Negl Trop Dis. 2023 Nov 27;17(11):e0011087. doi: 10.1371/journal.pntd.0011087 (PMC10703399; doi:10.1371/journal.pntd.0011087)
Supplement: Attachment — Submitted filename: PNTD_Ospina2023_review.pdf [file pntd.0011087.s003.pdf]

Review of "Effects of human mobility on the spread of Dengue in the region of Caldas, Colombia."

April 19, 2023

The authors use a metapopulation compartmental model to assess the effects of various mobility restrictions on the spread of a vector borne disease. The authors specifically focus on the case of dengue fever in the department of Caldas Colombia and how limiting movement between municipalities within the department affects the number of cases that develop. The paper is well written and could be potentially very useful to policy makers and other researchers. The authors show many scenarios where mobility restrictions are both effective and ineffective and examine the changes overall and at smaller levels. For these reasons, I really enjoyed the paper. However, I do have some fairly major concerns about the modeling, and I think the authors need to do a better job of linking their models to previous approaches.

1 Major Comments

Firstly, I believe the authors need to do a better job of linking their models to previous approaches. Equations (1) and (11) should have some citations. How are models (1) and (11) similar/different from other models in the literature? The assumptions before Equation (1) should also have some citations. Where did these assumptions come from and are they common in the literature? Do the assumptions make sense in Caldas? None of these important points are covered in the paper currently.

Most of my major comments are concerns about the modeling. Firstly, the authors state in Section 2.1 "The size of the human population N_h is assumed to be constant". However, for this to be true requires,

$$\frac{dN_h}{dt} = \frac{dS_h}{dt} + \frac{dI_h}{dt} + \frac{dR_h}{dt} = 0$$
$$\implies \Lambda_h = N_h \delta_h.$$

Broadly, this means the mortality rate needs to be roughly equal to the birth rate (or the rate of entry into the susceptible compartment). This is a very common assumption necessary to achieve a constant population in these models, see Keeling and Rohani (2007) Chapter 2 Section 2.1.2. However, the authors give Λ_h as $N_h \frac{1}{74.64*365}$, based on public health records, where as δ_h is taken from DANE statistics and differs between municipalities. Therefore, $\Lambda_h \neq N_h \delta_h$ currently and the population is not fixed. The solution is very easy however, just let $\Lambda_h = N_h \delta_h$ and keep δ_h at where it is currently.

Another concern is how temperature is modeled. The authors use $\beta(\overline{T})$, and so on, where $\overline{T}$ is the average temperature in the municipality. Doesn't it make more sense to use $\beta\big(T(t)\big)$ where $T(t)$ is the temperature at time t? I know the temperature doesn't vary a lot in Colombia across time so maybe this doesn't matter that much. However, $\beta\big(T(t)\big)$ feels like the natural way to model this effect, so it should be at least mentioned.

Finally, I have some concerns about the metapopulation model in Equation (11). My understanding is that the term

$$\beta^i \lambda_h^i S_h^i \sum_{j=1}^{n} \Upsilon_{ij} \frac{I_m^j}{N_{he}^j}$$

is meant to represent people who live in area i and are infected in areas $j = 1, ..., n$. If they are infected in area j then why is the biting rate in area i and the likelihood of a person becoming infectious in area i relevant? Shouldn't this term be

$$S_h^i \sum_{j=1}^{n} \Upsilon_{ij} \beta^j \lambda_h^j \frac{I_m^j}{N_{he}^j}?$$

2 Minor Comments

2.1 Introduction

1. On page 2 give a quick explanation about the difference between the SIR-SI and Ross-Macdonald models. As someone only passively familiar, I thought Ross-Macdonald did separate the humans and mosquitoes, so it is not immediately clear what the difference is.

2. On page 2, "that favor the eradication of the virus in humans population in a population". The end of the sentence does not make sense.

3. At the end of page 2, "and, evidently, it is essential to foster...". I do not think evidently is a good word choice here since it means something obvious and thus feels unscientific.

4. At the end of page 2 elaborate very briefly on the Singapore example. It is not clear what happened in Singapore and why it is relevant.

5. At the beginning of page 3 elaborate on why the expansion of urban environments makes the study of mobility in the spread of disease more important. I do not think it is obvious.

6. On page 3, "In scenario, the entire network...". There is a typo.

7. On page 3, "The first measure is to allow residents to leave but prevent outsiders from entering.". Entering and leaving what? It should be in the sentence.

8. On page 3, "was found to be the most effective measure,...". Was found to be the most effective measure among what? That information should be included. I find the beginning of this paragraph quite confusing, and I believe it needs revision.

9. On page 3, "The manuscript rounds off in section 4". I would say "concludes in Section 4".

2.2 Section 2.1

1. On page 4, "Susceptible humans can only get infected trough...". Should be through not trough.

2. On page 4 I would define the incubation period for the less technical audience.

3. On page 4, "There is not super-infection for either humans or mosquitoes.". What is a super infection?

4. On page 5 I do not think "Parameter estimation" is the right term. Normally parameter estimation refers to finding parameters through fitting the model to data.

5. On page 5, "the vital dynamics of the vector when $S_m = 0$,...". Do you mean when $\frac{dS_m}{dt} = 0$? In the next paragraph the authors introduce N_m without defining it. Is N_m supposed to be S_m^*?

6. For the x-axis in Figure 2, I would write [days] not [d].

2.3 Section 2.2

1. Regarding the mobility matrix Υ. First Equation (8) should read $\Upsilon_{ij} = \ldots$. However, I do not believe it is correct. It should be $\Upsilon_{ij} = .9$ if $i = j$ and $\Upsilon_{ij} = .1 * \frac{W_{ij}}{\sum_{l=1}^{n} W_{il}}$ if $i \neq j$.

2. I believe Υ_{ij} is just the fraction of people who live in area i but are travelling in area j. I would just state it plainly like this, so it is easier to understand.

2.4 Section 3.1

1. Figure 5 requires extensive revision. The x-axis is labeled t [d] which I do not know what that means. Is the x-axis supposed to be the distance between the nodes, what are the units? The y-axis is labeled I_h, but I_h is the incidence at a specific point in time. Is the y-axis supposed to be the total number of individuals who ever become infected during the epidemic (this is called the final size of an epidemic)? Why are the points in the graphs connected by lines that do not quite go through the points? The caption should read "as the distance between patches increases".

2. Looking at Figure 5, it does not seem like the total is reduced very much. It looks more like the cases just get redistributed. The Caldas example is much more convincing than this one, so I would investigate why the reduction in total cases is so much lower here.

3. The tables are introduced in the order 5, 3 and 2. Shouldn't they be introduced in numerical order?

2.5 Section 3.2

1. Regarding Figure 6, R^2 is not the correlation. It is the proportion of the variation in the dependent variable that is predictable from the independent variable(s). The authors should study a reference on R^2 to make sure it is interpreted correctly. Revise the caption as it has several grammatical errors. Finally, there should be a border around the map.

2. On page 11, "To calibrate the model to capture the number of dengue cases historically occurred in the department of Caldas (CO) during some outbreaks in the last decade, periodic perturbations in the number of infected mosquitoes during two rainy seasons in a year were introduced.". This is way too unclear. It seems like a big modeling change. An explanation with equations needs to be given.

3. On page 11, "The model was adjusted by minimizing the error between simulated and real cases.". What was adjusted? The perturbations? This and the perturbations require a lot more explaining.

2.6 Section 3.3

1. How were the mobility restrictions implemented in the model. I am guessing by setting W_{ij} and/or W_{ji} to 0? It might be worth mentioning this.

2. Figure 7 is quite confusing on its own. I think an important issue is that it is not clear how many cases there are without interventions, so the meaning of -150 for example is not clear. If there were 10,000 cases absent intervention then -150 is not a lot but for 1000 it is sizable. I would try to include information on the cases absent interventions in the figure. Also, perhaps the y-axis should read "Change in dengue cases"? I like the word "change" much more then "variation".

3. It would be interesting to see what happens when mobility is restricted in all five at once. Maybe add an extra column to Figure 7?

2.7 Section 3.4

1. I am wondering if Table 8 should be a graph, maybe a map, since it seems quite important.

2. On page 13, "The column (variation of cases) shows positive values when the mobility restriction reduces dengue cases and negative values when there is an inverse effect.". Positive should mean more cases and negative should mean fewer cases, compared to no interventions. Please flip it around.

3. On page 13, "Conceic˜ao in his study conducted in Sao Paulo". It is not just his study.

4. In the Supporting Information there are some parameters in the tables that are not defined, from what I can see, in the text. What is Λ_m min and Λ_m max in Table 3? What does Rain and Dryness mean in Table 4? Please look over these tables and ensure everything is defined in the text.

2.8 Conclusion

Regarding "Based on the results, it is recommended that health experts and administrative authorities limit mobility to areas with high incidence of the disease during dengue outbreaks in order to prevent its spread.". The authors mean the "Access" intervention right, but people can still leave? I agree looking at Figure 7 since at worst "Access" has no effect and usually it reduces the cases. But I do not know how many readers will pick up on that, so it might be worth elaborating on just a bit.

References

Keeling, M. J. and Rohani, P. (2007) *Modeling Infectious Diseases in Humans and Animals*. Princeton: Princeton University Press, illustrated edition edn.
