# Supplementary material for: Effects of human mobility on the spread of Dengue in the region of Caldas, Colombia
Source: PLoS Negl Trop Dis. 2023 Nov 27;17(11):e0011087. doi: 10.1371/journal.pntd.0011087 (PMC10703399; doi:10.1371/journal.pntd.0011087)
Supplement: Attachment — Submitted filename: ResponsesToReviewers.pdf [file pntd.0011087.s004.pdf]

Responses to the Reviewer's Comments and Description of Changes in the Manuscript

Dear Reviewers,

We sincerely appreciate your valuable comments and suggestions on our manuscript titled "Effects of human mobility on the spread of Dengue in the region of Caldas, Colombia." We have carefully considered each of the points raised and have made the following modifications to the manuscript:

- Model corrections: As per your feedback, we have made changes to the mathematical equations used in the model. These adjustments have been made to address the concerns raised and ensure the model's accuracy and reliability.
- Repetition of simulations: Due to the aforementioned changes in the model, we have repeated the simulations to obtain updated and accurate results. The revised findings corroborate that the effect of human mobility on the spread of Dengue depends on the disease's incidence in each location.
- Figure and table improvements: We have revised the figures and tables as per your recommendations to enhance their coherence with the presented data and provide a clearer representation.
- Writing enhancements: The manuscript's writing has been improved to enhance its readability and overall coherence.

Please find below the response to each of the comments raised by the referee in which we highlight in red the changes made to the manuscript.

We dare to hope that these modifications have adequately addressed the concerns raised by the reviewers and have enhanced the quality of the manuscript.

Once again, we sincerely appreciate your time and dedication in reviewing our work.

Yours sincerely,

Carolina Ospina (on behalf of all the authors).

Reviewer 1

1. **Please consider explaining why the simulation time of 400 days was chosen.**

   Actually all simulations are performed with a 365 days period. For the sake of clarity, we have included the following paragraph at the end of section 2.1.1 (page 6):

   We chose a 365 *days* period for the simulations because we had access to yearly dengue case records. This allowed us to train our model using a gradient descent method, where the cost function was the error between the simulated one-year incidence of dengue cases and the actual reported cases from local health authorities.

2. **Is there any stabilization when running the model for some more years?, dengue presents seasonality associated with temperature and rainy seasons, this was considered on the model (page 11), but it is not possible to observe it clearly on the general modeling results (Fig. 5), maybe at the final of the simulation. This effect seems to be present on the likelihood of a person becoming infectious (Fig.1 d). Maybe running for some more years this could become evident. Also, consider that a seasonal pattern could be present at holidays when people move across different regions.**

   The proposed model demonstrates stability when running the simulation for more than two years. In Figure 5, it is not possible to observe the increase in dengue cases during rainy seasons, as this figure displays the total dengue cases in a year for each of the analyzed nodes when increasing the distance between neighboring nodes.

   For clarity, the Figure 9 added in the annexes provides evidence of noticeable peaks in dengue infections coinciding with rainy seasons.

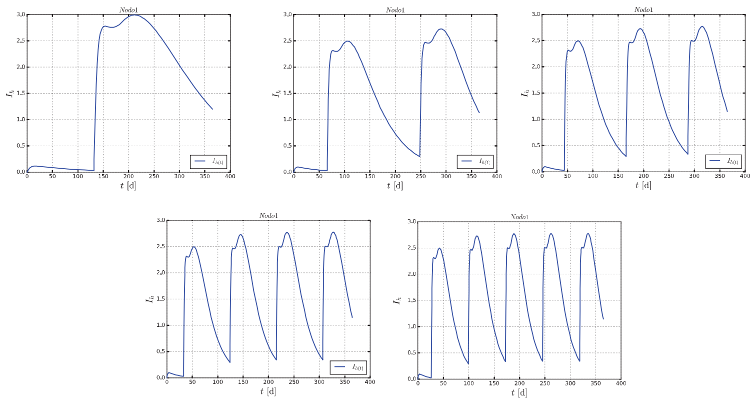

Fig 9. In a span of one year, this figure presents the total number of dengue infections in a node when one, two, three, four, and five rainy seasons occur.

While dengue behavior is indeed associated with temperature, our model does not incorporate temperature variations during the simulations. Instead, temperature values are used to establish parameters specific to each node, resulting in varying mosquito biting rates and mosquito mortality rates. Consequently, while the likelihood of a person becoming infectious remains

consistent, the biting rate of mosquitoes increases while their mortality rate decreases across different nodes. We do not increase the number of travelers in our simulations during non-rainy seasons; however, this is an aspect that can be explored in future work.

3. **Page 6. Section 2.1. Parameters are time dependent, consider including the time scale used. I suppose they are days.**

   The parameters described by equations (3),(4),(5) and (6) actually depend on temperature rather than time. In this work, the annual average temperature is used as an approximation to calculate these parameters when applying this model. Once defined, it is assumed that these parameters remain constant over time for subsequent simulations.

   For the sake of clarity, the following sentence has been added at the end of Section 2.1.1 (page 6):

   By using a daily time scale, we were able to capture the finer dynamics of dengue transmission and accurately represent the temporal patterns of the disease. The annual average temperature is used for fitting a model to a specific locality, and once the parameters are calculated using the procedure described in this section, they remain constant throughout the simulation time. This approach improved the clarity and accuracy of our model.

4. **Page 6. Section 2.2. Which are the considerations or references behind the assumption that 10% of the people are moving to another area and which is the time frame of the assumption.**

   This assumption is based on estimated traveler data provided by terrestrial transportation hubs. The specific time frame of this assumption was determined based on the available data at 2015.

   For clarity, we added the following sentence at the beginning of Section 2.2 (page 7): This assumption of 10% is based on the average number of daily travelers between the different municipalities of the Department of Caldas, as derived from information gathered at municipal terminals.

5. **Page 6. Figure 1c and 1f. Consider moving the legend from above the result.** We replaced Fig. 10 with Fig. 11.

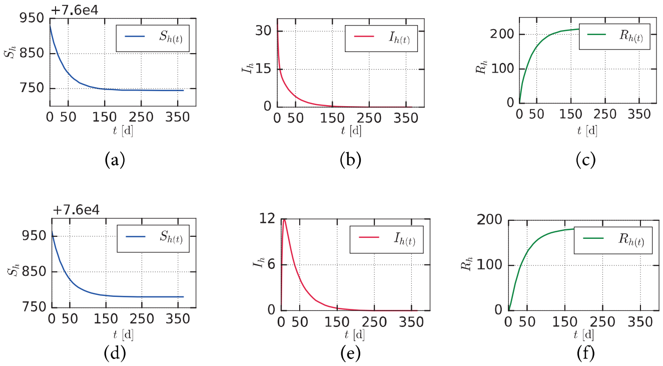

Fig 10. Behavior of the human population over time in the SIR-SI model. (a,c) Susceptible, (b,e) Infected and (c,f) Recovered. When $\rho < 1$ (a, b and c), and $\rho > 1$ (d,e and f).

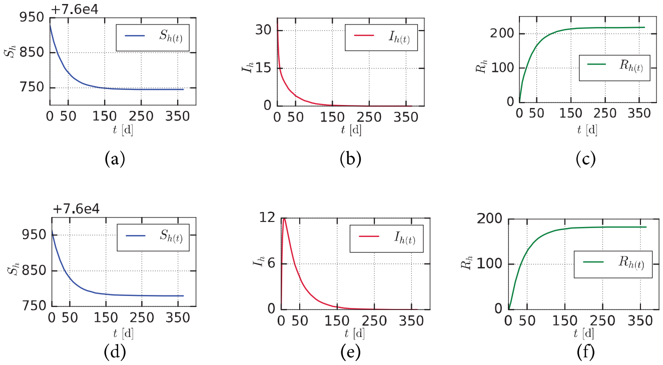

Fig 11. Behavior of the human population over time in the SIR-SI model. (a,c) Susceptible, (b,e) Infected and (c,f) Recovered. When $\rho < 1$ (a, b and c), and $\rho > 1$ (d,e and f).

6. **Equation 11. Please consider checking if in the compartments ${S_m}^i$, ${i_m}^i$,${S_H}^i$ and ${I_h}^i$, the notation "he" was defined previously. I suppose it is the human mobility flows integration, e from effective population. This could be added to the parameters table.**

   In equation 11, (N_{he}) represents the effective population, which refers to the actual number of individuals present in any given patch. (I_{he}) denotes the effective number of infected individuals in each patch i. To address this issue, we have included these terms in parentheses before each definition as follows:

   Agents' movements between patches i and j cause a redistribution of the population across the system; therefore it is necessary to adjust the equations (1) to account for the actual number of people that are in any patch i at any given time. In particular, the effective population (N_{he}) of patch i is defined as:

   $$N^i_{he} \quad = \sum_{j=1}^{n} \Upsilon_{ji} N^j_h \ , \tag{12}$$

   which accounts for the distribution of the residential population $\vec{N}$ and the mobility patterns of the individuals of the metapopulation $\boldsymbol{\Upsilon}$. Likewise, mobility also changes the effective number of infected (I_{he}) individuals in each patch i, which now reads:

   $$I^i_{he} \quad = \sum_{j=1}^{n} \Upsilon_{ji} I^j_h \tag{13}$$

7. Page 8. **On Fig. 3 please consider increasing the differentiation thickness of lines (ik, ki), (ki, kj) (ji ij) (kj jk) or try a combination of color patches to get an even better understanding of the meta-population dynamics.** Following the referee's suggestions, we have improved Figure 3 to better illustrate metapopulation dynamic.

8. **Page 10. Figure 5c. It is not possible to see the total number of cases.** We thank the referee for this comment. We replaced Figure 12 with Figure 13.

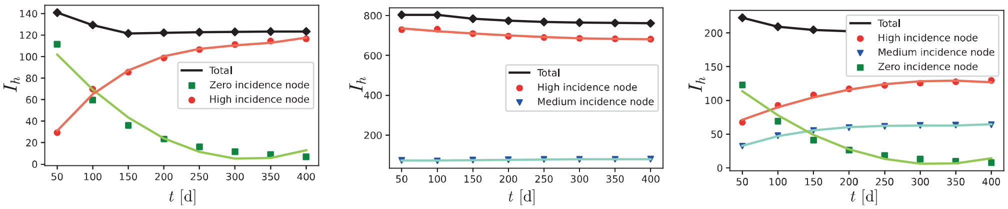

(a) (b) (c)

Fig 12. Evolution of dengue cases in patches as the distance increases when connecting a) High and a zero incidence node. b) High and medium incidence node. c) High, medium and zero incidence node. The green line shows the dengue cases in the zero incidence node, the blue line shows the cases in the medium incidence node and the red line shows the cases in the high incidence node. The black line is used for the total number of cases.

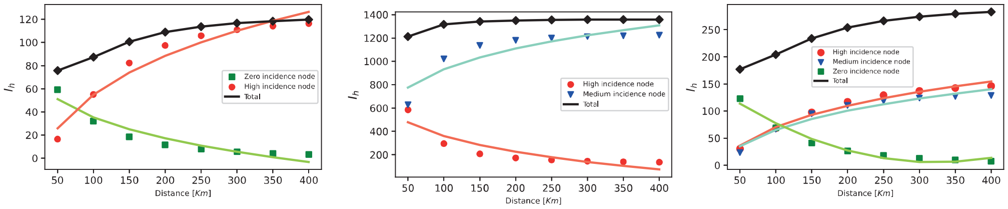

(a) (b) (c)

Fig 13. Evolution of dengue cases in patches as the distance increases when connecting a) High and a zero incidence node. b) High and medium incidence node. c) High, medium and zero incidence node. The green line shows the dengue cases in the zero incidence node, the blue line shows the cases in the medium incidence node and the red line shows the cases in the high incidence node. The black line is used for the total number of cases.

9. **Page 10. section 3.1.3 space is lacking in "locally,the situation".**

   On Page 10, in section 3.1.3, a space has been added between the comma and the word "the" to address the lack of space in the phrase "locally, the situation".

10. **Table 5 how the prob were calculated.** To enhance the clarity of the document regarding the calculation of the matrix Υ, modifications have been made to the initial paragraph of Section 3.2. The procedure, as described in Section 2.2, outlines the steps taken to calculate Υ. The population data used in the calculation was sourced from official records of the DANE, while the distances between municipalities were obtained through Google Maps. These updates aim to provide a comprehensive understanding of the methodology employed in the study.

    The interconnections between municipalities are determined based on road distances, forming the Υ matrix that governs the transition rates between pairs of municipalities. Notably, this matrix does not distinguish between municipalities within Caldas and those from neighboring departments that share close proximity and interconnectedness. The specific transition rates for all 33 municipalities are detailed in Table 5. The procedure employed to compute the

Υ matrix can be found in Section 2.2. Population data utilized for these calculations is sourced from authoritative records of the DANE, while distances between municipalities were obtained through Google Maps.

11. **Table 6. Consider indicating that this is a model prediction of Dengue at full connection.**

    The caption of Table 6 is changed by:

    Total number of dengue fever cases in the municipalities of Caldas obtained through the calibration of the proposed model.

12. **Page 11, last paragraph please consider rephrasing for better understanding: "The agreement be- tween real cases and those obtained after the SIR-SI model has been calibrated is shown in Fig.".**

    We have replaced: The agreement between real cases and those obtained after the SIR-SI model has been calibrated is shown in Fig. 6. In particular the map on the left shows the dengue cases reported by the health entities and on the right the dengue cases estimated with the model. It can be seen at the bottom that there is a correlation of 0.76 between the data estimated with the model and the cases occurred in the department of Caldas during 2015

    By: To validate the model, we conducted a comparison between the spatial distribution of reported cases in the department of Caldas during 2015 and the predictions obtained from our equations after calibrating the parameters (see Fig. 6). The model's predictions exhibit a strong positive correlation with the actual reported cases, yielding a Pearson correlation coefficient of $\rho_P = 1$. This high correlation demonstrates that the model effectively reproduces the spatial distribution of dengue cases in the department of Caldas during 2015.

Reviewer 2

- **Firstly, I believe the authors need to do a better job of linking their models to previous approaches. Equations (1) and (11) should have some citations. How are models (1) and (11) similar/different from other models in the literature?**

  The system of equations (1) in this study was formulated by combining two existing models from the literature. The first model, proposed by Bailey in 1975 [64], used for dengue models that address the transmission dynamics of a single serotype of the virus. The second model, developed by Sepulveda in her doctoral thesis [21], focuses on optimal control strategies to reduce dengue in the city of Cali, Colombia. By integrating the model (1) with the metapopulation formalism introduced by Soriano-Paños et al. [48, 49] for recurrent human mobility flows, the system of equations (11) is generated. After combining the works of Sepulveda and Soriano-Paños et al. to generate a new metapopulation model, another contribution of this work to the literature was to adjust the model with real values corresponding to the municipalities in the department of Caldas. This adjustment enabled us to simulate the cases of dengue that occurred during 2015.

  We have tried to better clarify the connection between previous works in the literature and ours in the revised version of the manuscript along the new introductory parts of sections 2.1 and 2.2.

- **The assumptions before Equation (1) should also have some citations. Where did these assumptions come from and are. They common in the literature? Do the assumptions make sense in Caldas? None of these important points are covered in the paper currently**

  As mentioned earlier, the assumptions related to environmental conditions were based on previous studies published in the literature that specifically address the spread of Dengue in Colombia. On the other hand, the remaining assumptions, which pertain to population dynamics and the compartmental model, are commonly made when applying the Ross-Macdonald model to study the spread of vector-borne diseases. These assumptions are fundamental for creating a simplified representation of the disease transmission process in a population.

  By combining these various assumptions and parameters, we were able to construct a comprehensive SIR-SI model that captures the essential features of Dengue transmission in the department of Caldas. Through simulations and calibration, the model demonstrated its ability to reproduce historical dengue cases. Nevertheless, it is important to continuously refine and validate the model as new data and insights become available to improve its accuracy and applicability in addressing public health challenges related to Dengue outbreaks.

  As stated in the previous point, the assumptions dependent on the environmental conditions were made based on previous studies published in the literature addressing the spread of Dengue in Colombia. The rest of the assumption, which are inherent to the population dynamics or the compartmental model, are usually made when using the Ross-Macdonald model to study the spread of vector-borne diseases [43].

  At the beginning of section 2.1 we have enumerated the assumptions and discussed them by linking with the previous apporaches to dengue transmission dynamics as discussed above.

- **Most of my major comments are concerns about the modeling. Firstly, the authors state in Section 2.1 "The size of the human population Nh is assumed to be constant"...**

  We thank the referee for raising this point. Let us remark that this assumption does not affect our results, as the time scale associated with population changes is much longer than the time scale considered in the simulations. Nonetheless, we agree with the referee that, unless fixing $\Lambda_h = N_h \delta_h$, the population size cannot be strictly assumed as a constant variable. Consequently, we have decided to remove this assumption from the description of the model not to mislead the reader.

- **Another concern is how temperature is modeled. The authors use β(T), and so on, where T is the average temperature in the municipality. Doesn't it make more sense to use β (T(t)) where T(t) is the temperature at time t? I know the temperature doesn't vary a lot in Colombia across time so maybe this doesn't matter that much. However, β (T(t)) feels like the natural way to model this effect, so it should be at least mentioned.**

  In the studies conducted by [12, 46, 52, 65, 66], the estimation of mosquito parameters (β), (λ_m), (λ_h), (δ_m) is performed using a single temperature value, either the average daily temperature or the average daytime temperature. As the referee mentioned, temperatures in the municipalities of Caldas do not vary significantly. Therefore, we made use of the average temperature to estimate the aforementioned parameters.

  As requested by the referee in page 12 we have mentioned that the use of the average temperature is based on the slight variations of it along the year.

- **Finally, I have some concerns about the metapopulation model in Equation (11). My understanding is that the term $\beta^i \lambda_h^i {S_h}^i \sum_{j=1}^{n} \Upsilon_{ij} \frac{{I^j}_m}{N_{he}^j}$ is meant to represent people who live in area i and are infected in areas j = 1, ..., n. If they are infected in area j then why is the biting rate in area i and the likelihood of a person becoming infectious in area i relevant? Shouldn't this term be**

  We thank the referee for this comment. As the referee correctly states, the contagion term should account for both the biting rate and the probability of infection at the destination of each individual.

  We have corrected the equations, calibrated again the model and re-run the analysis concerning the impact of acting on population mobility for the spread of Dengue in the department of Caldas. The results are qualitatively similar to those obtained in the previous version of the manuscript.

Minor Comments

Section 2.1

1. **On page 2 give a quick explanation about the difference between the SIR-SI and Ross-Macdonald models. As someone only passively familiar, I thought Ross-Macdonald did separate the humans and mosquitoes, so it is not immediately clear what the difference is.**

   We replaced: Alternative approaches for the study of the transmission of DENV include the Ross-Macdonald epidemic model [21]

By: Alternative approaches to the study of DENV transmission include the Ross-Macdonald epidemic model [21] where the gradual acquisition of immunity in humans is ignored.

2. **On page 2,"that favor the eradication of the virus in humans population in a population". The end of the sentence does not make sense.**

   We replaced: In all these approaches, the main goal is to define control strategies that favor the eradication of the virus in humans population in a population

   By: In all these approaches, the main goal is to define control strategies that favor the eradication of the virus in human populations.

3. **At the end of page 2, "and, evidently, it is essential to foster...". I do not think evidently is a good word choice here since it means something obvious and thus feels unscientific.**

   We replaced: For the particular case of dengue transmission, mobility determines the degree of exposure to the disease vectors [30, 31] and, evidently, it is essential to foster local dengue outbreaks in low incidence areas through the importation of cases from distant and high incidence regions [32, 33].

   By: For the particular case of dengue transmission, mobility determines the degree of exposure to vectors [30, 31]. Therefore, it is essential to avoid local dengue outbreaks in low incidence areas through the importation of cases from distant and high incidence regions [32, 33].

4. **At the end of page 2 elaborate very briefly on the Singapore example. It is not clear what happened in Singapore and why it is relevant.**

   we replaced: A remarkable recent example of such phenomenon was the reintroduction of the dengue virus in Singapore [34].

   By: A remarkable recent example of such phenomenon was the reintroduction of the dengue virus in Singapore [34] where dengue was successfully controlled, although the effects were not sustainable in the long term given the large movement of people and trade material in the dengue-endemic region.

5. **At the beginning of page 3 elaborate on why the expansion of urban environments makes the study of mobility in the spread of disease more important. I do not think it is obvious.**

   We replaced: The contribution of human mobility in the spread of dengue cases has become an essential factor given the expansion of urban environments and the increased frequency of international travels [27–29]. It has been projected that by 2030 more than half of the world's population will reside in urban areas in the tropics, due to population growth and migration from rural areas [36].

   By: The contribution of human mobility to the spread of dengue cases has become an essential factor given the increasing frequency of international travel and the expansion of urban environments [27, 28]), since, in the vast majority of cases, it is people who create artificial breeding sites for the vector that spreads the disease [26, 35]. It is projected that by 2030, over half of the world's population will reside in urban areas of the tropics, due to population growth and migration from rural areas [36].

6. On page 3, "In scenario, the entire network...". There is a typo. In scenario, the entire network is connected and two types of mobility restrictions are analyzed

   We replaced: Equipped with this framework, we focus on the analysis of how mobility constraints affect dengue transmission considering two control scenarios. In the first scenario, mobility constraints are concentrated in the nodes with the highest incidence. In the first scenario, the entire network is connected and three types of mobility restrictions are analyzed.

   By: Equipped with this framework, we aim to analyze how mobility constraints affect dengue transmission using two control scenarios. In the first scenario, we focus on limiting mobility in areas with the highest dengue incidence. As for the second scenario, we explore three types of mobility restrictions in a fully connected network.

7. **On page 3, "The first measure is to allow residents to leave but prevent outsiders from entering.". Entering and leaving what? It should be in the sentence.**
   On page 3, "was found to be the most effective measure,...". Was found to be the most effective measure among what? That information should be included. I find the beginning of this paragraph quite confusing, and I believe it needs revision.

   We replaced: The first measure consists of allowing residents to leave a municipality but preventing outsiders from entering. In this scenario, preventing outsiders from entering and allowing residents to leave a high-incidence municipality was found to be the most effective measure, resulting in a 22.51% reduction in the number of dengue cases in the entire region. The second is to allow outsiders to enter a high-incidence municipality, but not to allow its residents to leave. And finally isolation consists of preventing residents from leaving and outsiders from entering a municipality. The second scenario analyzes the effect of confinement, *i.e.*, totally restricting mobility throughout the network, preventing visitors from entering and residents from leaving a municipality. When isolation is applied, a reduction of cases of 42.67% is obtained compared to the unrestricted case

   By: The most effective measure, resulting in a 25% reduction in the number of dengue cases in the entire region, is to allow residents to leave a municipality but prevent outsiders from entering. Another measure involves allowing outsiders to enter a municipality with a high incidence of dengue but restricting the departure of its residents. Lastly, we implemented isolation by preventing both residents from leaving and outsiders from entering a municipality. In the second scenario, the effect of confinement is analyzed, which involves completely restricting mobility throughout the network, prohibiting visitors from entering and residents from leaving a municipality. Surprisingly, with isolation applied, there is an increase of 2.36% in dengue cases compared to the unrestricted scenario.

8. **On page 3, "The manuscript rounds off in section 4". I would say "concludes in Section 4".**

   We replaced: The manuscript rounds off in section 4 with a brief discussion of the results and their implication for informing public health decision makers.

   By: The manuscript concludes in section 4 with a brief discussion of the results and their implication for informing public health decision makers.

section 2

1. **Regarding the mobility matrix Υ. First Equation should read $\Upsilon = \frac{W_{ij}}{\sum_{l=1}^{n} W_{il}}$ However, I do not believe it is correct. It should be: $\Upsilon_{ij} = 0.9$ if $j = i$ and $\Upsilon_{ij} = 0.1 * \frac{W_{ij}}{\sum_{l=1}^{n} W_{il}}$ if $j \neq i$.**
   We acknowledge the reviewer for pointing out this important typo. In the revised version *we replace:*

$$\Upsilon = \frac{W_{ij}}{\sum_{l=1}^{n} W_{il}}$$

   By:

$$\Upsilon_{ij} = \begin{cases} 0.9 & j = i, \\ 0.1 \frac{W_{ij}}{\sum_{l=1}^{n} W_{il}} & j \neq i \ . \end{cases} \tag{14}$$

2. **I believe Υ_{ij} is just the fraction of people who live in area i but are travelling in area j. I would just state it plainly like this, so it is easier to understand.**

   We agree with the reviewer and in the revised version we replace: For this purpose, let us assume that each individual has his/her residence located inside a given patch but can move to other destination, identified as the workplace for instance. In the absence of data, we assume these movements to be governed by a fully-connected weighted matrix Υ.

   By: To represent the mobility flows, we introduce a matrix Υ in which each element, Υ_{ij}, denotes the fraction of the population living in patch i and traveling to patch j. Due to a lack of specific data, we assume these movements to follow a fully-connected weighted matrix Υ.

Section 3.1

- **Figure 5 requires extensive revision. The x-axis is labeled t [d] which I do not know what that means. Is the x-axis supposed to be the distance between the nodes, what are the units? The y-axis is labeled Ih, but Ih is the incidence at a specific point in time. Is the y-axis supposed to be the total number of individuals who ever become infected during the epidemic (this is called the final size of an epidemic)? Why are the points in the graphs connected by lines that do not quite go through the points? The caption should read "'as the distance between patches increases".**

  In the original graph, we made the mistake of labeling the x-axis as time in days, when in fact the points on the x-axis represent distances in kilometers between the analyzed nodes. The y-axis represents the total number of infected individuals in each node, depending on the distance between them. We applied an exponential fit to demonstrate the upward or downward trend in each node, which is why the points appear connected by lines.

  In the revised version we have replaced Figure 14 with Figure 15.

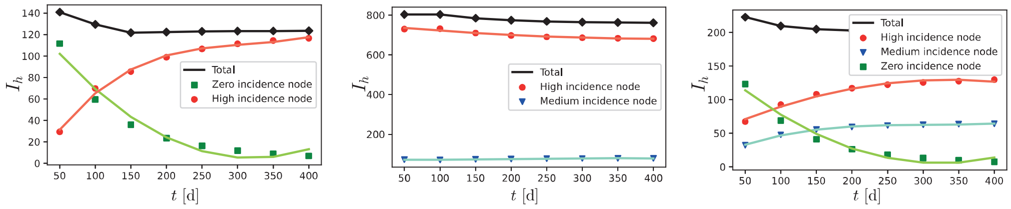

(a) (b) (c)

Fig 14. Evolution of dengue cases in patches as the distance increases when connecting a) High and a zero incidence node. b) High and medium incidence node. c) High, medium and zero incidence node. The green line shows the denge cases in the zero incidence node, the blue line shows the cases in the medium incidence node and the red line shows the cases in the high incidence node. The black line is used for the total number of cases.

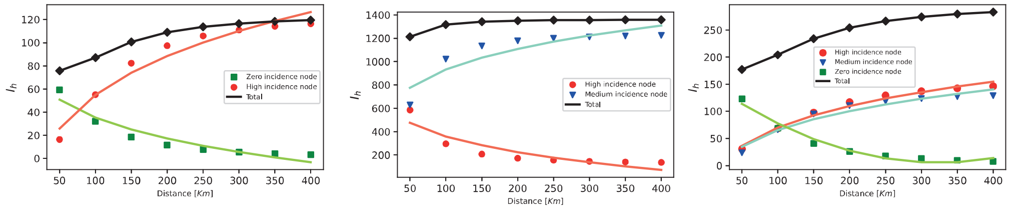

(a) (b) (c)

Fig 15. Evolution of dengue cases in patches as the distance increases when connecting a) High and a zero incidence node. b) High and medium incidence node. c) High, medium and zero incidence node. The green line shows the denge cases in the zero incidence node, the blue line shows the cases in the medium incidence node and the red line shows the cases in the high incidence node. The black line is used for the total number of cases.

- **Looking at Figure 5, it does not seem like the total is reduced very much. It looks more like the cases just get redistributed. The Caldas example is much more convincing than this one, so I would investigate why the reduction in total cases is so much lower here.**

  Upon analyzing Figure 5, it may appear that the total number of cases is not significantly reduced. Instead, it seems that the cases are redistributed among the nodes. However, it's worth noting that in all three scenarios, the black line representing the total cases exhibits a decreasing trend. While there is a redistribution of cases, there is indeed a reduction in the overall number of cases. The total cases are calculated by summing up the cases in the analyzed nodes.

- **The tables are introduced in the order 5, 3 and 2. Shouldn't they be introduced in numerical order?**

  The tables in the document were rearranged to ensure that they appear in an orderly manner when cited.

Section 3.2

- **Regarding Figure 6, R2 is not the correlation. It is the proportion of the variation in the dependent variable that is predictable from the independent variable(s). The authors should study a reference on R2**

to make sure it is interpreted correctly. Revise the caption as it has several grammatical errors. Finally, there should be a border around the map.

We thank the referee for this comment. We agree that our explanation of this coefficient was a bit sloppy as we were using the fact that, when performing linear regression on data, the coefficient of determination R^2 corresponds to the square of the Pearson correlation coefficient ρ_P. To avoid misleading explanations, we now show in the figure the Pearson correlation coefficient between both variables.

In particular, *we replace:* On the left, cases of dengue fever occurred in the department of Caldas during one year (2015). On the right, dengue cases predicted by the model during the same year. Below correlation between both variables.

By: Top row: Spatial distribution of the cases of Dengue reported in the department of Caldas in 2015 (Left panel) and that predicted by our model (Right panel). Bottom row: Cases predicted by the model as a function of the reported data in 2015 for each municipality in the department of Caldas. ρ_P indicates the Pearson correlation coefficient between these variables. The dashed grey line represent the perfect agreement scenario between model and real data.

- **On page 11, "To calibrate the model to capture the number of dengue cases historically occurred in the department of Caldas (CO) during some outbreaks in the last decade, periodic perturbations in the number of infected mosquitoes during two rainy seasons in a year were introduced.". This is way too unclear. It seems like a big modeling change. An explanation with equations needs to be given.**

  We agree with the referee that the original paragraph was unclear. In the revised version of the manuscript *we replace:* For the numerical simulations, the parameters β, λ_h, λ_m and δ_m are calculated with the procedure mentioned in section 2.1.1 and shown in Table 6 for each of the analyzed municipalities. The estimations are based on the average temperature value in each of them (see Table 7). In turn, the recruitment rate for humans is calculated with the value of life expectancy ($EVH = 74.64$), defined in public health records whereas humans mortality rate is obtained from DANE statistics.
  With the demographic, epidemiological and mobility parameters, initial conditions were set for each municipality (presented in Table 8). To calibrate the model to capture the number of dengue cases historically occurred in the department of Caldas (CO) during some outbreaks in the last decade, periodic perturbations in the number of infected mosquitoes during two rainy seasons in a year were introduced. As a necessary simplification, we only considered one dengue serotype; this assumption implies that individuals can only be infected once. The model was adjusted by minimizing the error between simulated and real cases. In particular, the SIR-SI simulation model with the 33 municipalities connected after calibration yields 1099 cases of dengue in Caldas (see Table 9).

  By: For the numerical simulations, we simplified the model by considering only one dengue serotype, assuming that individuals can be infected once. The key parameters, β, λ_h, λ_m, and δ_m, were individually calculated for each municipality using the method outlined in the "Estimated SIRSI Parameters" section (see Section 2.1.1). Let us note that in these estimations we have used the average temperature of each municipality (see Table 6). Although one can

use the time series of the temperatures recorded along the year, the slight variations of it in the region of Caldas allow us to make such simplification.

To account for the impact of the rainy seasons in Caldas, which occur twice a year, we introduced periodic perturbations to our model. These perturbations represent an increase in the number of infected mosquitoes during the two rainy seasons, modifying the values of infected mosquitoes for each municipality from $I_{mDrynees}$ to I_{mRain} (see Table 8). This calibration was aimed at capturing the historical dengue case numbers during outbreaks in the department of Caldas (CO) over the past decade, considering the well-established relationship between rainfall and dengue fever cases [35, 46, 51–54].

The recruitment rate for humans was determined using the life expectancy value ($EVH = 74.64$) from public health records, while the mortality rate for humans was obtained from DANE statistics. Through model adjustment, we minimized the error between simulated and real cases. The calibrated SIR-SI simulation model, with all 33 municipalities interconnected, resulted in 1033 dengue cases in Caldas (see Table 9).

- **"The model was adjusted by minimizing the error between simulated and real cases.". What was adjusted? The perturbations? This and the perturbations require a lot more explaining.**

  The adjustment made in this study involved independently varying the parameters β, λ_h, λ_m, and δ_m by ∓ 0.1 to minimize the error between the simulated and real cases. These parameters play a crucial role in the model's dynamics and directly influence the transmission and progression of dengue. By systematically modifying these parameters within a specific range, the model was fine-tuned to achieve the best fit with the observed data. The purpose of this adjustment was to optimize the model's accuracy in capturing the patterns and trends of dengue transmission, allowing for more reliable predictions and assessments of control measures.

Section 3.3

- **How were the mobility restrictions implemented in the model. I am guessing by setting Wij and/or Wji to 0? It might be worth mentioning this.**

  The mobility restrictions implemented in this work consisted of setting the rows or columns of the W matrix corresponding to the municipalities under mobility controls to zero. By assigning a value of zero to the entries in the matrix that represent mobility rates between the affected municipalities, the population flow between these nodes is prevented, effectively restricting their mobility completely. This allows us to assess how these restrictions impact the spread of dengue in the network of municipalities. For clarity, the following sentence has been added at the end of section 2:

  Let us finally note that the mobility restrictions implemented in this work consisted of setting the rows or columns of the W matrix corresponding to the patch under mobility controls to zero. By assigning a value of zero to the entries

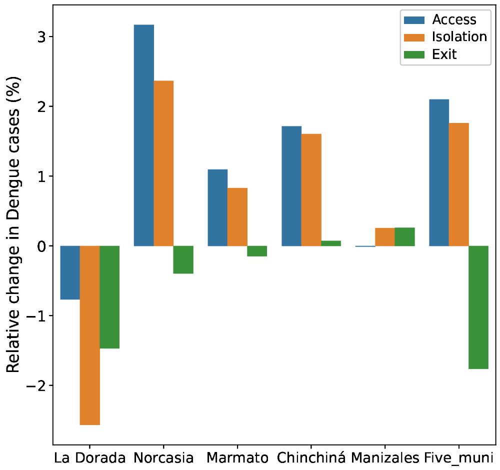

Fig 16. Variation of dengue cases in the department Caldas according to mobility restrictions when: i) no foreigners are allowed to enter the municipality (blue), no inhabitants can leave the municipality (green), the municipality is totally isolated (orange).

in the matrix that represent mobility rates between the affected patch, the population flow between these nodes is prevented, effectively restricting their mobility completely.

- **Figure 7 is quite confusing on its own. I think an important issue is that it is not clear how many cases there are without interventions, so the meaning of -150 for example is not clear. If there were 10,000 cases absent intervention then -150 is not a lot but for 1000 it is sizable. I would try to include information on the cases absent interventions in the figure. Also, perhaps the y-axis should read "Change in dengue cases"? I like the word "change" much more then "variation".**

  We agree with the referee that the changes in absolute terms does not provide valuable information in the absence of information about the total number of cases in the baseline scenario. To improve the presentation of the figure, we now represent the relative change in Dengue cases induced by each of the policies here explored, what gives a more realistic picture of the relevance of the different measures for the control of Dengue spread (see Fig. 16)

- **It would be interesting to see what happens when mobility is restricted in all five at once. Maybe add an extra column to Figure 7?**

  In Figure 7, a sixth column is added to display the relative changes in dengue cases in the department when applying mobility restrictions to all five municipalities collectively. This column shows the overall impact of the combined restrictions on the total number of dengue cases in the department, providing valuable insights into the effectiveness of these measures when implemented together.

Section 3.4

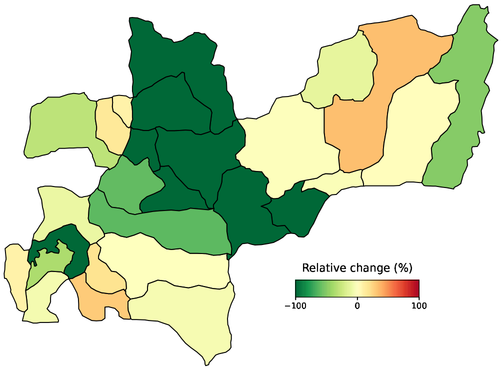

Fig 17. Variation of dengue cases in the department of Caldas when quarantine are implemented. The green color indicates a reduction in the number of dengue cases, with darker tones representing more significant changes. Conversely, the red color symbolizes an increment in dengue cases after the quarantine are applied.

- **I am wondering if Table 8 should be a graph, maybe a map, since it seems quite important.**

  With the data from Table 10, we have generated a color map (see Fig. 17 wich is new Fig. 8 in the revised manuscript) that illustrates the variations in dengue cases when applying quarantine measures in the department of Caldas. The color map provides a visual representation of the changes in dengue incidence across different municipalities in response to the implementation of quarantine measures. The darker green tones indicate a reduction in dengue cases, while the red tones indicate an increase in cases when the quarantine is implemented. This map serves as a valuable tool for understanding the spatial distribution and impact of quarantine measures on dengue transmission in the department.

- **On page 13, "The column (variation of cases) shows positive values when the mobility restriction reduces dengue cases and negative values when there is an inverse effect.".Positive should mean more cases and negative should mean fewer cases, compared to no interventions. Please flip it around.**

  We have replaced: The column (variation of cases) shows positive values when the mobility restriction reduces dengue cases and negative values when there is an inverse effect.

  By: Negative values in the "variation of cases" column indicate a reduction in dengue cases due to mobility restrictions, while positive values indicate an inverse effect.

- **On page 13, "Conceic ao in his study conducted in Sao Paulo". It is not just his study.**

  We have replaced: The results obtained in this scenario coincide with those obtained by *Conceição* in his study conducted in Sao Paulo [56] where there was a reduction in dengue cases as a collateral result of population confinement to hinder the advance of COVID-19 pandemic

  By: Interestingly, these findings are aligned with those obtained by *Conceição et al.* [56] in a study conducted in São Paulo (Brasil), where a reduction in dengue

cases was observed as a collateral result of population confinement to hinder the advance of the COVID-19 pandemic.

- **In the Supporting Information there are some parameters in the tables that are not defined, from what I can see, in the text. What is Λ_m *min* and Λ_m *max* in Table 3? What does Rain and Dryness mean in Table 4? Please look over these tables and ensure everything is defined in the text.**

  In this work, the terms Λ_m *min* and Λ_m *max* are omitted from Table **??** as they are not used in the main text. For the terms Rain and Dryness, they are mentioned in Section 3.2. In particular, $I_{mDrynees}$ represents the number of infected mosquitoes during the dry season, while I_{mRain} corresponds to the number of infected mosquitoes during the rainy seasons.

Conclusions

- **Regarding "Based on the results, it is recommended that health experts and administrative authorities limit mobility to areas with high incidence of the disease during dengue outbreaks in order to prevent its spread.". The authors mean the "Access" intervention right, but people can still leave? I agree looking at Figure 7 since at worst "Access" has no effect and usually it reduces the cases. But I do not know how many readers will pick up on that, so it might be worth elaborating on just a bit.**

  We have replaced: The model presented in this study makes it possible to analyze the effect of applying control measures to reduce dengue cases in a region. By comparing the results of the model with the actual cases, it has been established that the estimated values have a realistic correspondence. Based on the results, it is recommended that health experts and administrative authorities limit mobility to areas with high incidence of the disease during dengue outbreaks in order to prevent its spread. This model can be extrapolated to any region, if the average temperature, altitude, number of inhabitants, human vital statistics and aedic records are known. Moreover, the mobility control measures here discussed can be taken into account for the development of public health policies in endemic regions.

  By: The model presented in this study makes it possible to analyze the effect of applying control measures to reduce dengue cases in a region. The comparison between the model's predictions (1099 cases) and the actual dengue cases (1033 cases) in the department of Caldas has shown a fair agreement that validates our model as a tool for evaluating different contention policies. To effectively mitigate the spread of dengue during outbreaks, it is recommended that health experts and administrative authorities implement mobility limitations in areas with a high incidence of the disease. The results, as illustrated in Figure 7, highlight the significant positive impact of complete restrictions on access to endemic municipalities, leading to a substantial reduction in overall dengue cases. The model's applicability can be extended to other regions by incorporating relevant aspects such as average temperature, altitude, population size, human vital statistics, and entomological records. Moreover, the proposed mobility control measures offer valuable insights for informing the development of targeted public health policies in endemic regions. Further research and collaboration are warranted to validate and refine these findings, paving the way for evidence-based strategies to effectively combat dengue transmission.
